# Advances in Imaging for Tricuspid Transcatheter Edge-to-Edge Repair: Lessons Learned and Future Perspectives

**DOI:** 10.3390/jcm12103384

**Published:** 2023-05-10

**Authors:** Francesca Romana Prandi, Stamatios Lerakis, Martina Belli, Federica Illuminato, Davide Margonato, Lucy Barone, Saverio Muscoli, Marcello Chiocchi, Mario Laudazi, Massimo Marchei, Marco Di Luozzo, Annapoorna Kini, Francesco Romeo, Francesco Barillà

**Affiliations:** 1 Division of Cardiology, Department of Systems Medicine, Tor Vergata University, 00133 Rome, Italy; martynebelli@gmail.com (M.B.); fe.illuminato@gmail.com (F.I.); lucy.barone82@gmail.com (L.B.); saveriomuscoli@gmail.com (S.M.); m_marchei@hotmail.com (M.M.); diluozzomarco@gmail.com (M.D.L.); francesco.barilla@uniroma2.it (F.B.); 2 Department of Cardiology, Mount Sinai Hospital, Icahn School of Medicine at Mount Sinai, New York, NY 10029, USA; stamatios.lerakis@mountsinai.org (S.L.); annapoorna.kini@mountsinai.org (A.K.); 3 Cardiovascular Imaging Unit, San Raffaele Scientific Institute, 20132 Milan, Italy; margonato.davide@hsr.it; 4 Department of Diagnostic Imaging and Interventional Radiology, Tor Vergata University, 00133 Rome, Italy; marcello.chiocchi@gmail.com (M.C.); mariolauda@gmail.com (M.L.); 5 Department of Departmental Faculty of Medicine, Unicamillus-Saint Camillus International University of Health and Medical Sciences, 00131 Rome, Italy; romeocerabino@gmail.com

**Keywords:** tricuspid valve, tricuspid edge-to-edge repair, structural heart interventions, valvular heart disease, cardiac imaging, echocardiography, cardiac CT, cardiac MRI

## Abstract

Severe tricuspid valve (TV) regurgitation (TR) has been associated with adverse long-term outcomes in several natural history studies, but isolated TV surgery presents high mortality and morbidity rates. Transcatheter tricuspid valve interventions (TTVI) therefore represent a promising field and may currently be considered in patients with severe secondary TR that have a prohibitive surgical risk. Tricuspid transcatheter edge-to-edge repair (T-TEER) represents one of the most frequently used TTVI options. Accurate imaging of the tricuspid valve (TV) apparatus is crucial for T-TEER preprocedural planning, in order to select the right candidates, and is also fundamental for intraprocedural guidance and post-procedural follow-up. Although transesophageal echocardiography represents the main imaging modality, we describe the utility and additional value of other imaging modalities such as cardiac CT and MRI, intracardiac echocardiography, fluoroscopy, and fusion imaging to assist T-TEER. Developments in the field of 3D printing, computational models, and artificial intelligence hold great promise in improving the assessment and management of patients with valvular heart disease.

## 1. Introduction

### 1.1. Tricuspid Valve Anatomy

**Figure 1:**
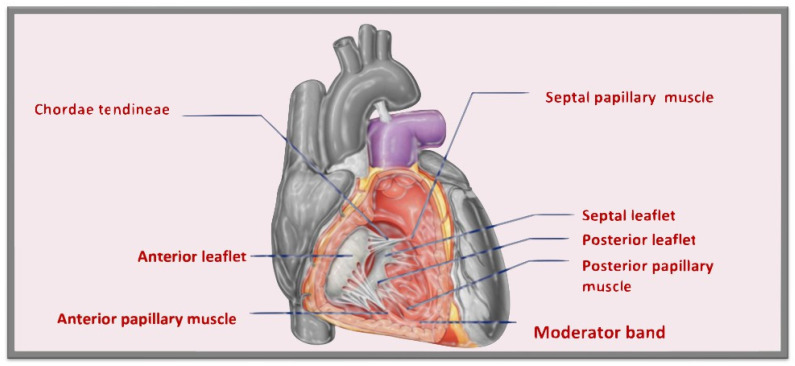
Anatomy of the tricuspid valve.

The tricuspid valve (TV) is the largest (its orifice area is between 7 and 9 cm^2^) and it is the most anteriorly and inferiorly positioned of the four cardiac valves [1]. The TV consists of a fibrous annulus, leaflets, and a tensor apparatus that includes chordae tendinae, papillary muscles, and adjacent right atrial (RA) and right ventricular (RV) myocardium (Figure 1).

The tricuspid annulus (TA) is an asymmetric, saddle-shaped ellipsoid that is dynamic, allowing it to change with different loading conditions. Its highest points are the anteroseptal part (near the RV outflow tract and the aortic valve) and the posterolateral part. The most apical point is the posteroseptal part (near the inflow of the coronary sinus) and the anterolateral segment. In healthy individuals, the circumference of the TA is 12 ± 1 cm and the area is 11 ± 2 cm^2^ [2,3].

The leaflets are usually semicircular or triangular and are basally attached to the fibrous annulus. The valve generally consists of three leaflets: anterior, posterior, and septal, although a study by Lama et al. showed that the number of TV leaflets can vary from three to seven, with accessory leaflets often being the smallest [4]; additional leaflets appearance may also be caused by deep folds in the leaflets. A bicuspid TV is another possible anatomic variant [5].

The anterior leaflet (or superior or infundibular) is the largest and most mobile leaflet and it is adjacent to the RV outflow. The septal leaflet (or medial) is usually the second largest leaflet and, under normal conditions, it inserts into the septum ≤ 10 mm apical to the septal insertion point of the anterior mitral leaflet, making it more apically displaced than the mitral valve. The posterior leaflet (or inferior) is usually the smallest of the three, it often has multiple scallops and it is the least mobile. However, a clear separation between the anterior and posterior leaflets is not always evident [6].

In the distal quarter to third of the leaflets are the insertions of the chordae tendineae, that are fibrous cords. The valve leaflets are attached by the chordae tendineae to the papillary muscles or, in contrast to the mitral valve, directly to the RV myocardial wall. Primary chordae are attached to the free edge of the valve leaflets and play an important role in preventing regurgitation. Secondary chordae, on the other hand, attach to the basal part of the ventricular surface of the valve.

The TV has three main papillary muscles. The anterior papillary muscle is often the largest, originates from the moderator band, and is located at the commissure between the anterior and posterior leaflets. The septal papillary muscle is usually the least prominent, it is often rudimentary and is absent in 20% of the population; sometimes, there can be more than one septal papillary muscle. The moderator band connects the anterior papillary muscle with the septal papillary muscle and contains the right bundle branch. The posterior papillary muscle is often bifid or trifid [5].

Accurate knowledge of the TV apparatus is crucial for transcatheter TV interventions planning. The TV also presents important surrounding structures, such as the noncoronary sinus of Valsalva, the right coronary artery, the coronary sinus ostium, the superior and inferior vena cavae, the atrioventricular node, and the right bundle of His [7].

### 1.2. Tricuspid Transcatheter Edge-to-Edge Repair

Approximately 4% of patients aged 75 years or over have clinically relevant tricuspid regurgitation (TR) [8]. Primary (or organic) TR is observed in <10% of cases and results from an anatomical abnormality of the TV, while secondary (or functional) TR represents > 90% of cases and is due to TA dilation and leaflet tethering caused by RV enlargement and dysfunction or enlarged RA due to chronic atrial fibrillation (AF). In functional TR, annular expansion is mainly along the anterolateral border in subjects with left heart disease and sinus rhythm, while it is mostly along the posterior border in the case of TR associated with AF.

Severe TR has been associated with adverse long-term outcomes in several natural history studies [9] and is indeed independently associated with impaired survival and worsening heart failure (HF). The management of severe TR is mainly pursued with optimal medical therapy and surgery. Isolated TV surgery is associated with high mortality and morbidity, both in-hospital and during follow-up when patients present late [10,11]. Transcatheter tricuspid valve intervention (TTVI) therefore represents a promising field and it may be currently considered according to the Heart Team at experienced valve centers in patients with severe secondary TR that are symptomatic, inoperable, anatomically eligible, and in whom a symptomatic or prognostic improvement can be expected after the procedure [12].

In a propensity-matched case-control study, TTVI in high-risk patients with symptomatic severe TR was associated with greater survival and reduced HF rehospitalizations at 1-year follow up compared with medical therapy alone [13]. Another propensity-match analysis confirmed that TTVI was associated with reduced one-year all-cause mortality compared to conservative therapy in patients with severe TR, and showed that it might exert its highest treatment effect in patients with mid-range reduced RV function [14].

Current TTVIs include several treatment options. Tricuspid transcatheter edge-to-edge repair (T-TEER) with the TriClip (Abbott Vascular, Santa Clara, CA, USA) or leaflet approximation using the PASCAL systems (Edward Lifesciences, Irvine, CA, USA) are the most frequently used as a result of their safety, ease of use, and availability [15,16]. They work in a similar way, generally bridging together the septal leaflet with the anterior leaflet (bicuspidization) through paddles and clasps; in addition, the PASCAL system also includes a central spacer, that aims to improve the TR by occupying the regurgitant orifice. FORMA (Edward Lifesciences, Irvine, CA, USA) is another coaptation device which is only represented by a spacer. Other transcatheter techniques include direct annuloplasty, heterotopic caval valve implantation, and TV replacement [17].

## 2. Pre-Procedural Evaluation for Tricuspid Transcatheter Edge-to-Edge Repair

### 2.1. Two-Dimensional and Three-Dimensional Transthoracic and Transesophageal Echocardiography

Echocardiography should be the first imaging modality used to evaluate TR [12], since it allows to confirm TR diagnosis and to assess its etiology, severity, prognosis, and, finally, to evaluate the feasibility of a specific intervention. Key features to evaluate for the pre-procedural planning of TV transcatheter repair are the location of the largest vena contracta and the motion and length of the tricuspid leaflets [18].

The complexity and the anatomical variability of the TV require multiple transthoracic windows for a comprehensive assessment, also because of the difficulty in visualizing all three leaflets in the same plane due to the large orifice area [1]; moreover, tricuspid leaflets are thinner than mitral valve leaflets, so they are more difficult to visualize [5].

**Figure 2:**
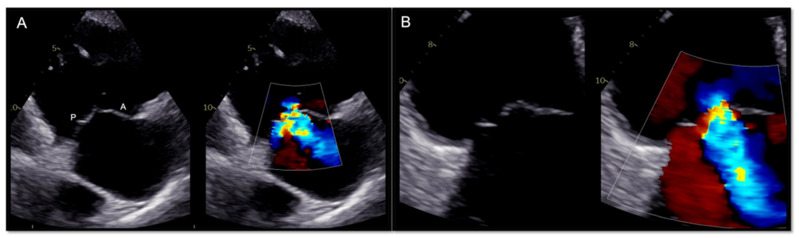
Transthoracic echocardiogram in two dimensions. Parasternal long axis RV-inflow view (**A**,**B**). Since the interventricular septum and the coronary sinus ostium are not visualized, the leaflets imaged are the posterior (P) and anterior (A); Color doppler documented severe functional tricuspid regurgitation (TR).

**Figure 3:**
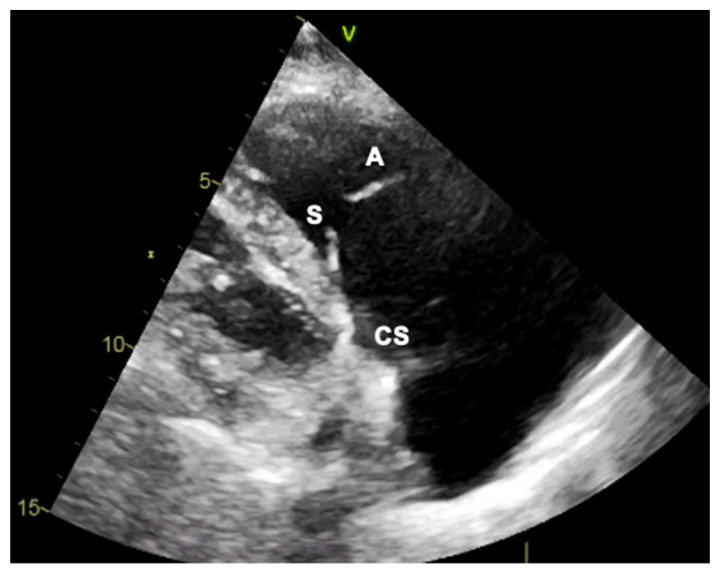
Transthoracic echocardiogram in two dimensions. If the muscular interventricular septum along with the coronary sinus (CS) ostium are seen, then the leaflets visualized are the septal (S) and the anterior (A).

**Figure 4:**
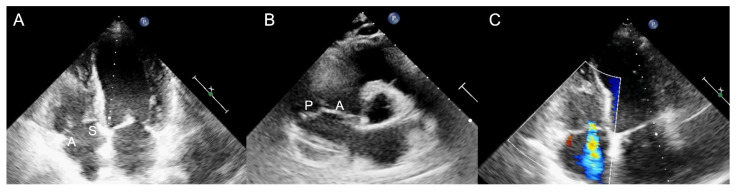
Transthoracic echocardiogram in two dimensions. (**A**) Apical four-chamber view: the septal leaflet (S) is seen adjacent to the septum and the anterior leaflet (A) adjacent to the right ventricular free wall; (**B**) Parasternal short-axis view: the anterior leaflet (A) is visualized next to the aorta and the posterior leaflet (P) on the opposite site, adjacent to the right ventricular free wall; (**C**) Apical four-chamber view with color-Doppler documenting tricuspid regurgitation (white arrow).

Generally, the transthoracic echocardiography (TTE) parasternal long-axis RV-inflow view detects the septal leaflet adjacent to the septum (if the interventricular septum (IVS) or the coronary sinus ostium are visualized) or the posterior leaflet (if IVS or coronary sinus ostium are not seen) and the anterior leaflet adjacent to the RV free wall (Figure 2 and Figure 3). The apical four-chamber view allows us to see the septal leaflet adjacent to the septum and the anterior leaflet adjacent to the RV free wall. In the parasternal-short axis view the posterior leaflet is seen adjacent to the RV free wall and the anterior or septal leaflet adjacent to the aorta (Figure 4) [19].

**Figure 5:**
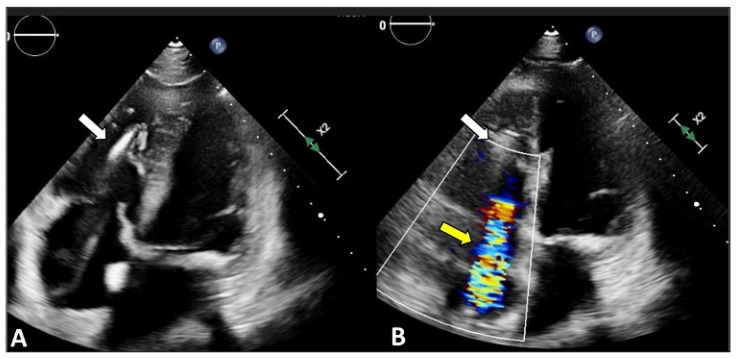
Cardiac implantable electronic device (CIED)-related tricuspid regurgitation (TR). Transthoracic echocardiogram, apical four-chamber view documents CIED RV lead (**A**,**B**, white arrows) and RA lead that cause relevant TR visible with Color-doppler (**B**, yellow arrow).

Two-dimensional (2D) TTE allows for an assessment of the pathophysiological mechanism of TR, as according to a new integrated classification by Pratz et al. in primary or organic (caused by leaflet structural abnormalities, the rarest), secondary or functional (atrial or ventricular dilatation, the most frequent), and cardiac-implantable electronic device (CIED)-related (leaflet impingement, rupture, adherence, or perforation) (Figure 5) [17].

Two-dimensional TTE also allows for an evaluation of the severity of TR. Severe TR may be identified through qualitative (large color flow jet area, dense continuous-wave doppler jet with early peaking), semi-quantitative (vena contracta width ≥ 7 mm, PISA radius ≥ 9 mm, systolic flow reversal in hepatic vein flow), and quantitative parameters (effective regurgitant orifice area (EROA) ≥ 0.40 cm^2^, regurgitant volume ≥ 45 mL). Recently, an extended classification to “massive” (vena contracta ≥ 14 mm, EROA ≥ 0.6 cm^2^) and “torrential” (vena contracta ≥ 21 mm, EROA ≥ 0.80 cm^2^) has been proposed [20].

Two-dimensional TTE also allows for left ventricle (LV) evaluation, RV assessment (RV size, function, and remodeling through strain analysis), and pulmonary hypertension (PHT) diagnosis and classification [17].

**Figure 6:**
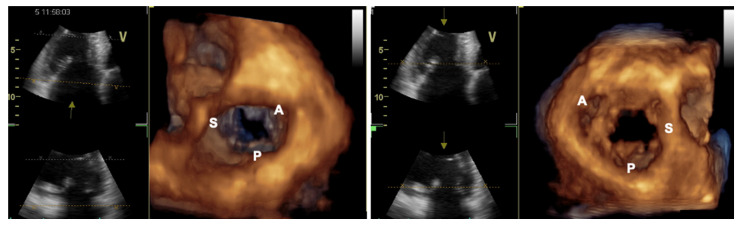
Transthoracic three-dimensional echocardiogram documenting tricuspid valve leaflets from atrial (**left panel**) and ventricular (**right panel**) perspectives. A = anterior leaflet, S = septal leaflet, P = posterior leaflet.

Three-dimensional TTE allows for an evaluation of all three TV leaflets at the same time (Figure 6) and to study the geometric interactions of the TV apparatus [21], the RV shape, and functional remodeling (3D-derived volumetric measures and 3D strain analysis) with a good correlation to cardiac magnetic resonance (CMR) measures [22].

The three-dimensional TTE TV acquisition protocol includes an apical four-chamber view with and without Color and a parasternal RV-inflow view with and without Color; the TV en face view should locate the septal leaflet and the interatrial septum in the 6 o’ clock position, the posterior leaflet to the left, and the anterior leaflet and aorta to the right [1,23]. To visualize a particular section of the TV, it is possible to adjust the cropping plane [23]. Three-dimensional TTE may offer equal or better quality than three-dimensional transesophageal echocardiography (TEE) because RV occupies an anterior position [1]. A limitation of three-dimensional TTE imaging is the lower temporal resolution compared with two-dimensional echocardiography [23].

These assessments are necessary for pre-procedural screening to select eligible patients for transcatheter TV repair, following the echocardiographic exclusion criteria of the CLASP TR easy feasibility study (severe LV dysfunction with LV ejection fraction (LVEF) < 30%, severe RV dysfunction, coaptation gaps > 10 mm, leaflet length < 8 mm), the TRILUMINATE PIVOTAL trial (severe LVEF < 20%, coaptation gap > 20 mm), or common for both of them (indications for left-sided or pulmonary valve correction, previous TV procedures, tricuspid stenosis, rheumatic leaflets degeneration, moderate-severe calcification in anulus, subvalvular apparatus or in the grasping area, Ebstein Anomaly, pulmonary artery systolic pressure > 70 mmHg, CIED that would interfere with appropriate placement of device). This first screening allows for the identification of the correct intervention timing and avoids futile procedures [15,16,24].

The anatomical assessment of the TV is better studied through two-dimensional (2D) and three-dimensional (3D) transoesophageal echocardiography (TEE), which is key to obtain a detailed functional valve anatomy, necessary to assess repairability. Anteroseptal jet location, a small septolateral gap (≤7 mm), and trileaflet TV morphology are the favorable anatomical features to perform leaflet approximation strategy; a septolateral coaptation gap between 7–8.5 mm and a posteroseptal jet location are considered feasible but suboptimal anatomical characteristics to perform [17]. A comprehensive TEE imaging protocol should be performed in order to choose the best treatment option according to the anatomical features and to evaluate if TEE imaging quality can adequately guide the interventional procedure [17]; for the latter, it is important to perform part of the TEE screening with the patient in a supine position to simulate the quality of the acoustic window during intervention. TEE also allows for a better identification of lead interference from CIED and the presence of a thrombus or mass that contraindicate any TV intervention.

**Figure 7:**
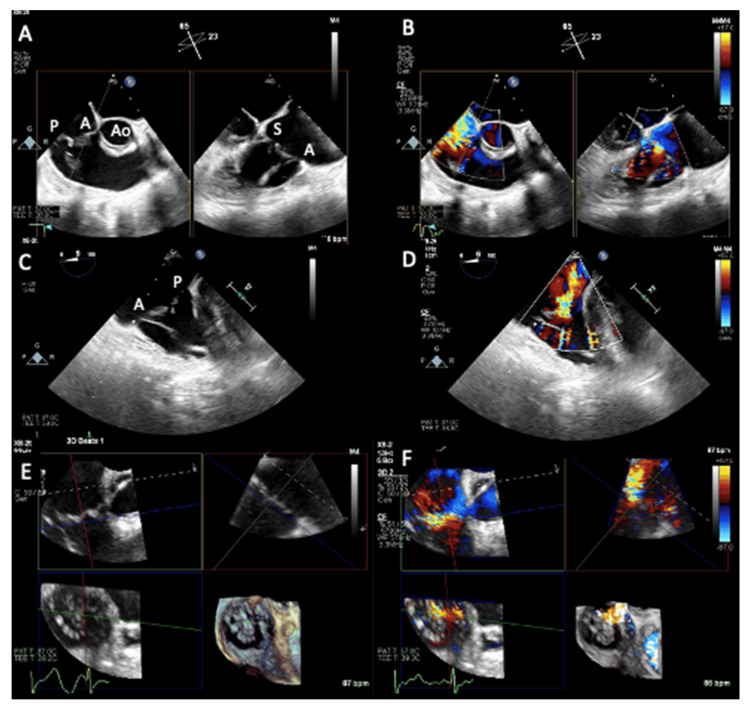
Transesophageal echocardiogram two-dimensional views (**A**,**B**): RV inflow-outflow view with biplane imaging; (**C**,**D**) deep esophageal 0° view) and multiplanar views (**E**,**F**) documenting flail anterior leaflet determining malcoaptation and severe tricuspid regurgitation with color Doppler (**B**,**D**,**F**). Ao = aorta, P = posterior leaflet, A = anterior leaflet, S = septal leaflet.

**Figure 8:**
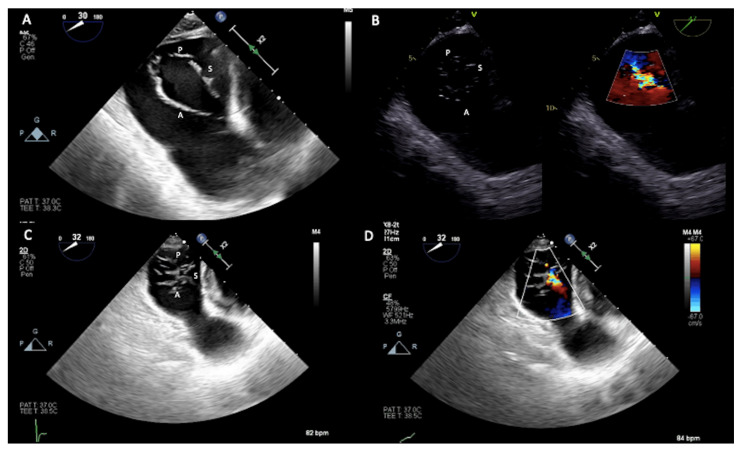
Two-dimensional transesophageal echocardiogram transgastric (TG) views. (**A**) TG short axis 30° documenting all three tricuspid valve leaflets in the open position during diastole; (**B**) in another patient, TG short axis 47° also with Color Doppler that documented functional tricuspid regurgitation due to leaflets tethering; (**C**) in another patient, TG short axis 32° also with color Doppler (**D**) with closed leaflets during systole documenting malcoaptation, especially between the anterior and septal leaflets, determining tricuspid regurgitation. The anterior leaflet (A) is in the far field, the posterior leaflet (P) is in the near field, and the septal leaflet (S) is on the right.

The main pre-procedural two-dimensional TEE views firstly include mid-esophageal (ME) views, where the TV is in the far field and the annular plane cannot be aligned perpendicular to the ultrasound beam. An ME four-chamber view at 0° allows for a visualization of the septal leaflet adjacent to the septum and the anterior or posterior leaflet on the other side, which can be clarified using biplane imaging. An ME RV inflow-outflow view at 50–60°, also defined as the “TV commissural view”, images the anterior leaflet adjacent to the aorta and the posterior leaflet opposed, and with biplane imaging we can visualize the septal leaflet as well (Figure 7). TV commissural view biplane imaging also allows us to see both posterior-septal and anterior-septal coaptations, in order to exactly define jet location. A deep-esophageal (DE) view at 0° visualizes the posterior or septal and on the other side the anterior leaflet, while a DE view at 60° sees the posterior and anterior leaflets and permits an alignment of the doppler beam of the regurgitant jet. Transgastric (TG) views allow us to see all of the leaflets at the same time from the TG RV inflow-outflow view at 0° using biplane imaging or a TG short-axis at 20–60° using single-plane view; in both views, we can visualize the anterior leaflet in the far field, the posterior leaflet in the near field, and the septal leaflet on the right (Video S1, Figure 8). TG views are crucial for the pre-procedural planning of tricuspid transcatheter repair by coaptation devices as they allow us to measure the coaptation gaps at the tip of the three leaflets and the exact location of the regurgitant orifice. Finally, a deep-transgastric (DT) view at 120–160° can be also performed for aligning the doppler across the TV regurgitant jet [25].

**Figure 9:**
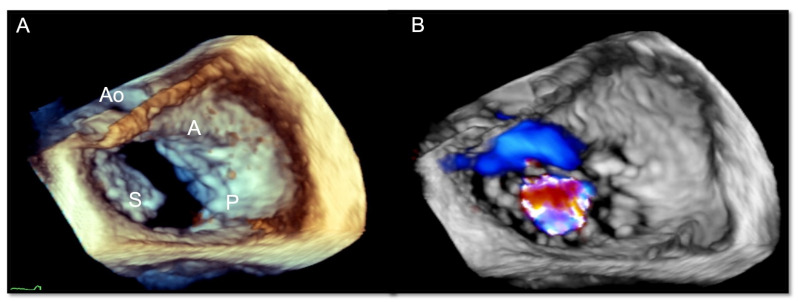
Transesophageal echocardiogram. (**A**) 3D en face imaging of the tricuspid valve acquired from a mid-esophageal commissural view with Z rotation: this is called the “surgeon’s view” and images the aorta and anterior leaflet (A) on top; (**B**) with Color Doppler documenting severe tricuspid regurgitation (TR). A = anterior leaflet, S = septal leaflet, P = posterior leaflet.

Considering the TV anatomic variability, 3D imaging is crucial for pre-procedural planning as it has higher accuracy in the identification of the number and location of the leaflets. Leaflets identification is based on the surrounding anatomy, with the aorta adjacent to the anteroseptal commissure and the coronary sinus adjacent to the posteroseptal commissure. A 3D en face TV view is acquired from the 2D commissural view and may be realized in two different orientations: with Z rotation (180° rotation) to obtain a “surgeon’s view” with aorta and anterior leaflet on top (Figure 9), or without Z rotation to obtain the “interventionalist’s view” with the aorta in the far field at 5 o’clock, the anterior leaflet in the far field next to the aorta, the posterior leaflet in the near field, and the septal leaflet on the right side, close to the interventricular septum. The interventionalist’s view is often preferred because it is similar to the TG short axis view, the only 2D view that simultaneously shows all three leaflets and measures the coaptation gap [25,26]. Furthermore, 3D multiplanar reconstructions (MPR) performed in real time or off-line may help to better define leaflet lengths (≥8 mm for a favourable clasping) and mobility, coaptation gaps, and the regurgitant orifice location to plan the intervention [17]. Leaflet insertion should be located ≥6 and ≥9 mm for NTR and XTR TriClip, respectively [27].

Three-dimensional echocardiography also has a prognostic role; indeed, impaired pre-procedural three-dimensional echocardiographic-derived RVEF ≤ 45% has been associated with increased all-cause mortality at 1-year follow up after tricuspid TEER [28].

### 2.2. Cardiac Computed Tomography

Although the relevance of cardiac computed tomography (CCT) imaging in patient screening and the pre-procedural planning of transcatheter TV treatments is less well established compared to echocardiography [29], it has high spatial resolution and offers the potential for detailed anatomic assessment, which can be critical to minimize procedural complications and to optimize results [30]. CCT is fundamental for transcatheter TV annuloplasty and valve replacement procedures, but it is not required for T-TEER [31,32]. CCT may overcome many of the limitations of 2D echocardiography by providing excellent anatomical and functional analysis of the TV apparatus, as well as its relation to the nearby structures. For example, CCT is helpful in the pre-procedural assessment of patients with a mechanical aortic prosthesis, where acoustic shadowing may limit precise TV visualization by echocardiography. Moreover, in patients who have had previous cardiac surgery, CT scan can assess the presence of any resultant distortion in cardiac anatomy.

**Figure 10:**
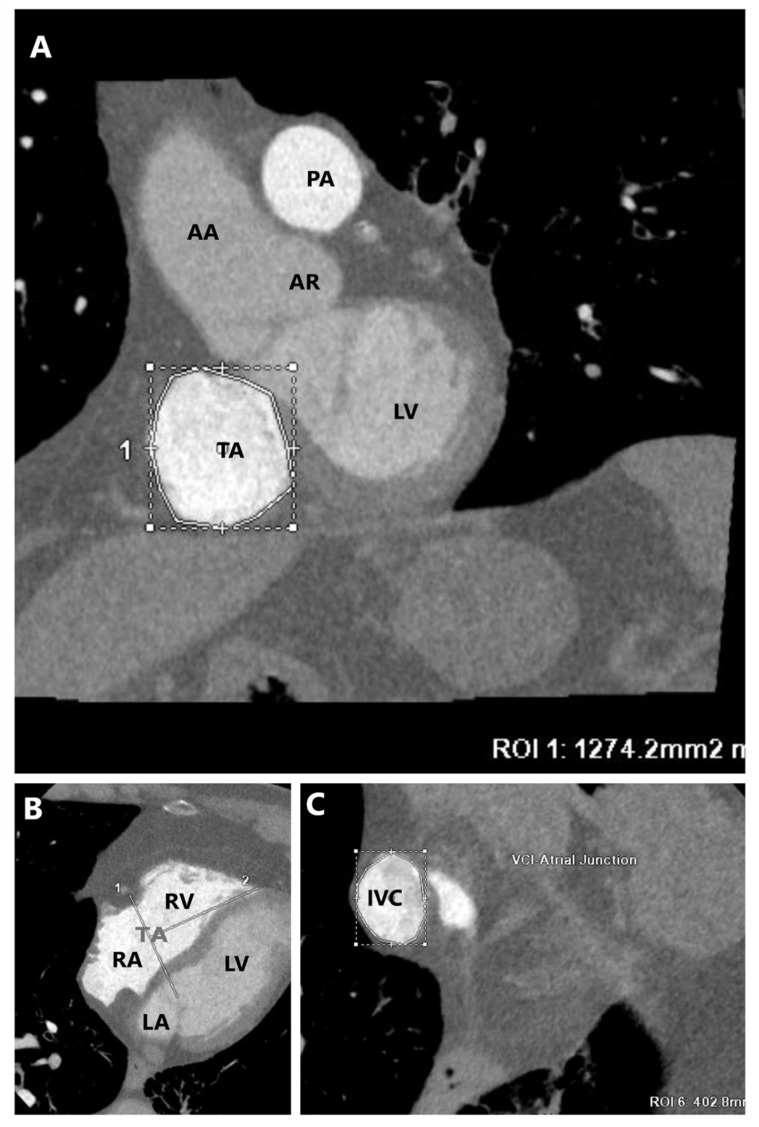
Assessment of TA dimensions by Cardiac Computed Tomography performed with a specific right–sided opacification protocol and by using multiplanar planes. (**A**) A planar cross-sectional area (short-axis) of TA that allows measurements of the TA area, perimeter, anteroposterior, and septal- lateral diameters. (**B**) Right-chambers morphology for the measurement of distance between the TA and the RV apex. (**C**) A transverse plane of the IVC. TA = tricuspid annulus, LV = left ventricle, AR = aortic root, AA = ascending aorta, PA = pulmonary artery, RA = right atrium, RV = right ventricle, LA = left atrium, IVC = inferior vena cava.

To maximize image quality, a specific CT acquisition protocol with right-sided opacification [33] is recommended (Figure 10), with triphasic contrast bolus admixture to avoid streak artifacts [34].

CCT may provide useful information on TV and leaflet anatomy, the valve gap, mechanisms of TR, and the best fluoroscopic angles for coplanar alignment and leaflet grasping strategy [32,34].

Using the multiplanar reconstruction, a short-axis plane can be reconstructed on the annular level starting from RV two- and four-chamber views [35]; this allows for an optimization of the tricuspid annular shape, calcification, and dimensions including the TA perimeter, area, anteroposterior, and septal-lateral diameters [7,17]; these measurements should be evaluated in both end-systole and mid-diastole because of the dynamic variability of the annular size [35]. Moreover, due to the complex non-planar saddle-shaped structure of the TA, the use of 3D semiautomated software can overcome the limitations of a 2D approach. CCT also assesses the relationships of the TA with the adjacent structures, especially the right coronary artery, although this is more relevant for annuloplasty procedures [17].

Additional MPR planes parallel to the TV can optimize the visualization of the TV leaflets anatomy and identification of a four-leaflet configuration variant, which is associated with an increased risk of residual TR, especially in patients with a wide coaptation gap or TR jet from the posteroseptal or anteroposterior commissures [36]. It also allows the measurement of the position and extent of the coaptation gap and TV leaflet tethering grade, including tenting height, angle, and area, which is always measured in mid-systole; by aligning the reformation planes with the narrowest portion of the regurgitant orifice during mid-systole, is possible to measure the anatomical regurgitant orifice area, which may represent a flow-independent anatomic parameter of TR severity [35,37,38,39].

CCT can also provide useful information on the right chambers, such as right ventricular and right atrial volumes [40], as well as target site definition, which are valuable in assessing the anatomic feasibility of a transcatheter therapy [7], and the RV function, including ejection fraction and stroke volume, has excellent reproducibility using CMR as a reference [41,42]. In patients with pacemaker/ICD leads that are not MRI compatible, CCT represents a useful alternative for right-chamber evaluation. CCT can also evaluate the interaction of the TV leaflets with eventual pacemaker/ICD leads when they are not clear on echocardiography [31].

Furthermore, CT can assess peripheral vessels in terms of suitability of access and route to the heart. Indeed, CT can be used to analyze the measurements of the inferior vena cava, as well as the size of the femoral veins, which are used to deliver the sheaths and the devices to their target [7,27].

Finally, CCT can predict the ideal fluoroscopic projections to be intra-procedurally used and generate projection images, which can be exported and used to support intraprocedural fluoroscopy with fusion imaging for guiding treatments, which is critical for reducing procedure times and radiation exposure [43].

X-ray exposure remains the main disadvantage of CCT, although the newest generation of CT scanners allow for substantially shorter scan times, lower radiation doses, and less iodinated contrast utilization [32].

### 2.3. Cardiac Magnetic Resonance

Cardiac magnetic resonance (CMR) is not the first line imaging modality, but given its excellent spatial resolution, it has an additive value to 3D echocardiography for both anatomic and functional assessment of the TV, TA, and right-sided chambers [30]; it can be particularly helpful in obese patients and in patients with breast implants and lung diseases. CMR also has the advantage of not using ionizing radiations. Its main limitations are cardiac arrhythmias and the presence of intracardiac leads that may create artifacts and compromise CMR image quality [32]; in addition, it is less accessible, and patients should be medically stable and not claustrophobic [44].

**Figure 11:**
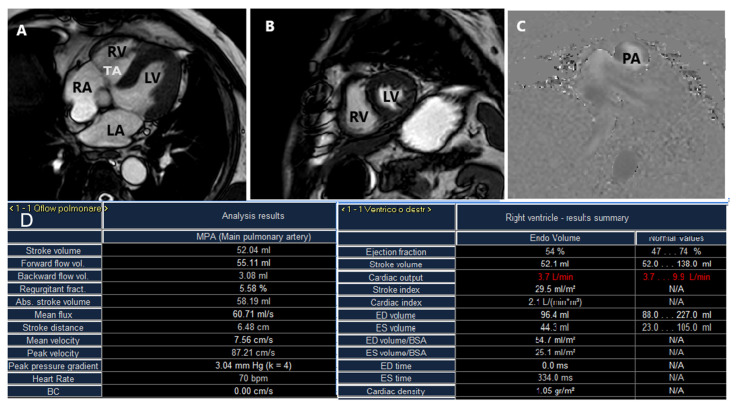
Cardiac magnetic resonance allows for the assessment of right ventricular (RV) function and tricuspid valve regurgitation by cine-imaging. (**A**) Four-chamber view of the heart. (**B**) Short-axis view of RV and LV. (**C**) Q-Flow sequences (example of pulmonary artery Q-flow assessment). (**D**) A typical analysis results table for Pulmonary Q Flow and RV function with important parameters for pre-procedural and post-procedural assessment. RA = right atrium, RV = right ventricle, LA = left atrium, LV = left ventricle, TA = tricuspid annulus, PA = pulmonary artery.

CMR is useful to assess the severity of regurgitant TV lesions in patients with inadequate echocardiographic quality or discrepant results; it represents the reference method for the evaluation of RV volumes and function allowing for an evaluation of the consequences of TR (Figure 11); finally, it allows for an assessment of myocardial fibrosis [12,29,45,46].

TR severity can be assessed by CMR using indirect (by calculating TR volume (TRV) as RV stroke volume- forward pulmonic flow volume or TR fraction (TRF) as TR volume/RV stroke volume ×100) or direct methods (by effective regurgitant orifice area measurement) [30]. No specific CMR cutoffs for TR severity have been established yet, but a regurgitant fraction ≥ 40% is typically considered to be hemodynamically significant [30]. Functional TR severity, as assessed by CMR imaging, represents an independent predictor of mortality, even after adjustment for clinical and imaging variables, including RV ejection fraction (RVEF); a TRV ≥ 45 mL or a TRF ≥ 50% have the highest risk of excess mortality under medical management [47].

Pre-procedural CMR-quantified RVEF and RV end systolic volume indexes were independently associated with increased postoperative cardiac death and major cardiac events in patients with isolated severe TR undergoing corrective surgery [48]. Baseline global RV dysfunction (RVEF ≤ 45%), as determined by the combined presence of both longitudinal and circumferential RV dysfunction using CMR in patients undergoing transcatheter TV repair, represented an independent predictor for the composite end-point of all-cause mortality and HF hospitalization [49].

CMR also allows for a non-invasive measurement of the pulmonary vascular resistance (PVR), which may help improve the selection of candidates for tricuspid interventions since patients with end-stage RV failure may present reduced pulmonary artery pressure but higher PVR [50].

Finally, myocardial fibrosis detection by CMR has a prognostic implication for RV failure and may be useful to define the optimal timing of interventions in patients with severe TR [17].

## 3. Intra-Procedural Guidance for Tricuspid Transcatheter Edge-to-Edge Repair

### 3.1. Two-Dimensional and Three-Dimensional Transesophageal Echocardiography

**Figure 12:**
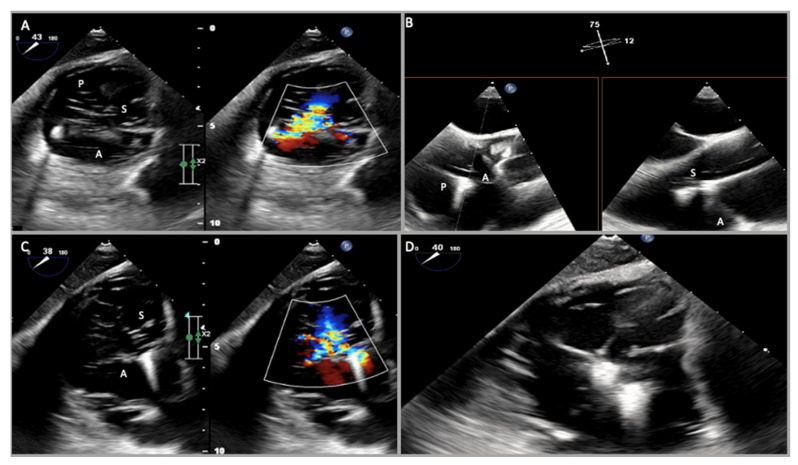
Intraprocedural monitoring by transesophageal echocardiography. (**A**) Transgastric short-axis view. Color Doppler is useful to guide positioning of the clip over the desired location relative to the regurgitant orifice. (**B**) Mid-esophageal commissural biplane view and (**C**) transgastric short-axis view: the position of the clip delivery system (CDS) with respect to the target lesion can be checked and corrected using 2D (single or biplane) and Color Doppler. (**D**) Transgastric short-axis view. The quality of leaflet grasp is verified by the limited leaflet mobility relative to the tips of the clip arms. A = anterior leaflet, S = septal leaflet, P = posterior leaflet.

**Figure 13:**
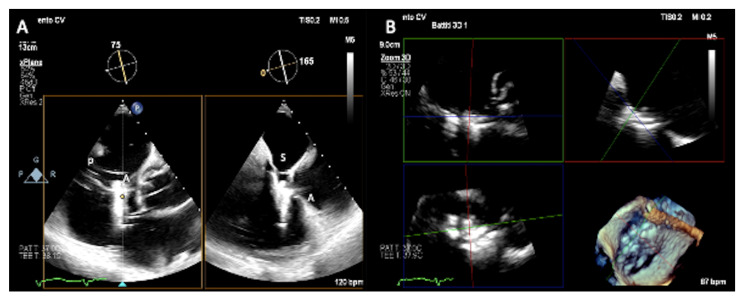
Intra-procedural monitoring by transesophageal echocardiography. (**A**) Right ventricular inflow-outflow view with biplane imaging to monitor septal (S) and anterior (A) leaflets grasping and (**B**) live 3D multiplanar reconstruction. A = anterior leaflet, S = septal leaflet, P = posterior leaflet.

**Figure 14:**
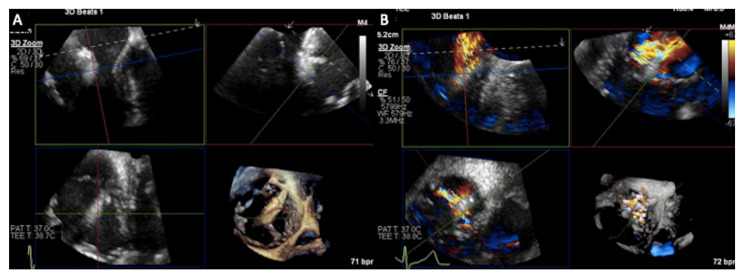
Intraprocedural transesophageal echocardiography. Multiplanar views and derived 3D reconstructions (**A**), and with color Doppler in (**B**) are crucial to help correct clip positioning.

ME and DE RV inflow/outflow views (TV “commissural” views) and TG views are the main views for the intraprocedural guidance of tricuspid transcatheter repair by coaptation devices (Figure 12, Figure 13 and Figure 14); the main procedural phases are represented by delivery system advancement, device orientation, grasping, pre-release evaluation, and post-release assessment.

The first step is represented by the introduction and advancement of the delivery system into the right atrium (RA), guided by the bicaval view with biplane imaging [51]. For the PASCAL system, it is important, after checking the delivery system tip position (1–2 cm into the RA from inferior vena cava), to monitor the safe introduction of the elongated device, avoiding trauma to the surrounding structures and to image implant closure and shortening, necessary to reduce the risk of injuries [26]. ME and DE RV inflow/outflow (TV “commissural” views) direct the delivery system toward the TV, carefully avoiding interatrial septum perforation, and guide the preliminary device position using color Doppler. TG short-axis view using color Doppler and 3D en face views are important to confirm implant and arms orientation and rotation along the commissure line and advance into the RV with continuous rotation monitoring [52]. The clasping phase (capture of leaflets) is mainly supported by the commissural view with biplane imaging (Figure 10), slightly adjusting the secondary plane toward the aorta in case of anteroseptal grasping or away from the aorta in case of posteroseptal grasping [51]. Furthermore, 3D imaging and multiplanar reconstructions can be used for the confirmation of adequate leaflets insertion [26,52]. When adequate tissue bridge, leaflets insertion, and implant position are defined, three assessments have to be checked before the device release through 2D and 3D commissural and TG views: residual TR severity (by color Doppler and by 3D color Doppler planimetry of the vena contracta), residual TV orifice area, and gradient (≤3 mmHg acceptable) and hemodynamic benefit through hepatic venous inflow. In this phase, prior to release, any repositioning can be performed. The same parameters have to be checked in post-release assessment. At this point, the possible need for a second implant in the same intervention must be assessed [26,51].

The role of TTE during the procedure is generally limited, since TEE allows us to continuously follow the procedure with less exposure of the structural imager to the radiations, although TTE may be useful in the case of an intraprocedural complication assessment, such as pericardial effusion [52].

### 3.2. Intracardiac Echocardiography: Strenghts and Limitations

The idea of using catheter-based devices to image the cardiac structures dates back to the 1960s [53], but the understanding of intracardiac echocardiography (ICE) potential in guiding structural interventional procedures was only proposed three decades later [54]. In recent years, the exponential development of several structural interventional cardiology procedures has strongly urged a better visualization of cardiac structures, with ICE representing an alternative/complementary intraprocedural imaging tool to conventional TEE, since it is able to provide high-resolution real-time visualization of cardiac structures, the continuous monitoring of the catheter location within the heart, and early recognition of procedural complications such as pericardial effusion or thrombus formation. The main advantage of ICE is that it can be performed by the primary operator of the interventional procedure under conscious sedation, since it does not require general anesthesia, while TEE, when it is performed in a supine patient, requires general anesthesia and oro-tracheal intubation to protect the airways; additionally, ICE eliminates the risk of esophageal trauma associated with TEE and reduces fluoroscopy exposure. Therefore, ICE may shorten the procedure time (mostly because of the avoidance of general anesthesia), decrease the costs and logistics of the procedure, eliminate the esophageal risks, facilitate more rapid patient recovery, and improve outcomes [55].

For intraprocedural guidance of TV transcatheter repair, ICE is particularly useful in patients with low quality TEE imaging [56], which is one of the exclusion criteria from enrollment in TV edge-to-edge trials [15]. Tricuspid leaflets visualization by TEE can be challenging due to the distance between the TEE probe and the anteriorly located tricuspid valve, especially in patients with right heart enlargement; other limitations are represented by acoustic shadowing due to left-sided implants, device catheters, and calcifications [57]. Conversely, ICE can be flexibly positioned within the right atrium near the TV, potentially addressing many of the current imaging challenges for tricuspid transcatheter interventions [57] and representing an appealing alternative to ensure co-axial alignment [58]. There are five main ICE probe locations: the mid right atrium, the low right atrium, the RV inflow, the RV outflow, and the left atrium [59]; to visualize the anterior and septal leaflet, the probe can be positioned in the mid right atrium, while for posterior and septal leaflet grasping, the probe is brought more towards the TV [60].

**Figure 15:**
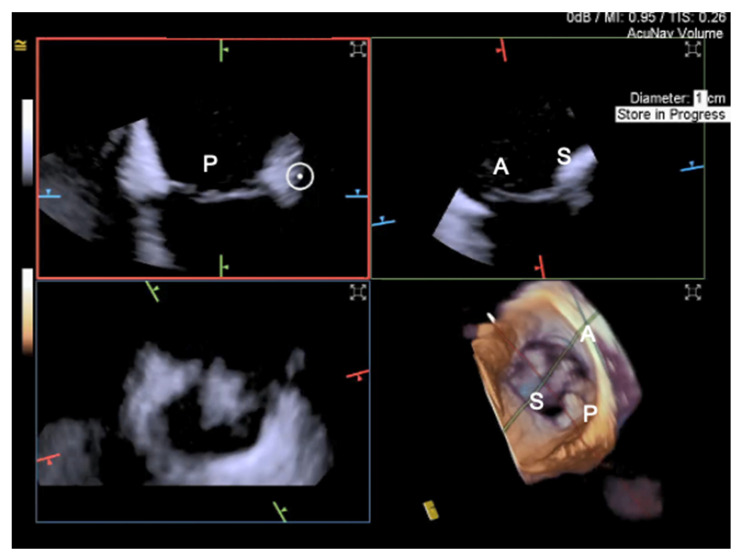
4D Volume ICE Multiplanar reconstruction of the tricuspid valve. The marker indicates the aorta. P = posterior leaflet; A = anterior leaflet; S = septal leaflet.

**Figure 16:**
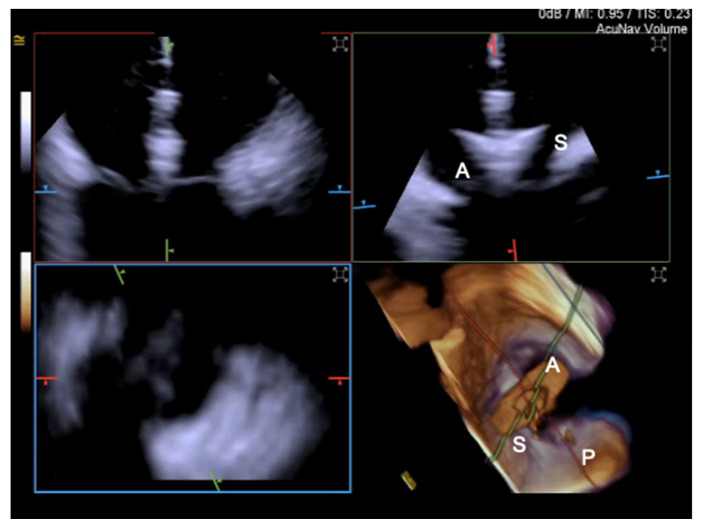
4D Volume ICE Multiplanar reconstruction of the tricuspid valve used to optimize clip trajectory. A = anterior leaflet; S = septal leaflet; P = posterior leaflet.

**Figure 17:**
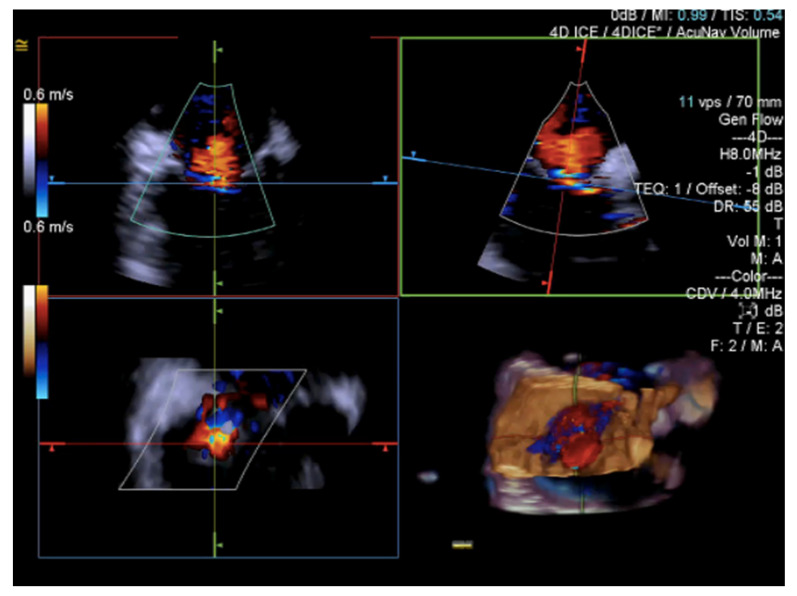
4D Volume ICE Multiplanar reconstruction of the tricuspid valve with color.

Until recently, the use of ICE for structural heart procedures has been limited by the presence of only 2D imaging planes, lower color Doppler quality, and volume size capability, and by the need for considerable catheter manipulations to visualize the target cardiac structures [61]. Enhancements in ICE technology have developed new 4D volume catheters that now allow biplane, 3D, and multiplanar reconstructions (Figure 15, Figure 16 and Figure 17), as well as co-registration with fluoroscopy; in addition, new catheters also present increased stiffness, that allows for a more stable catheter position [57].

A complementary approach using both TEE and ICE imaging with 4D catheters has been shown to be useful during T-TEER. Indeed, ICE can overcome TEE acoustic shadowing from the clip delivery system, facilitating leaflet grasping and confirmation of leaflet insertion and mobility [60], which may help reduce the incidence of procedure complications such as single-leaflet device attachment.

Despite the superb ability of ICE to visualize the tricuspid apparatus and the RV, current limitations for its use for intra-procedural guidance of tricuspid transcatheter interventions are represented by the presence of limited published data on guidance for TV interventions and by the operator learning curve; additionally, there is limited reusability for ICE catheters, with increased costs compared with TEE, and a higher risk of complications related to the additional access site and catheter manipulation. Finally, there is also a lack of standardization regarding ICE imaging acquisition, interpretation, reporting, and related society guidelines [59].

### 3.3. Fluoroscopy

**Figure 18:**
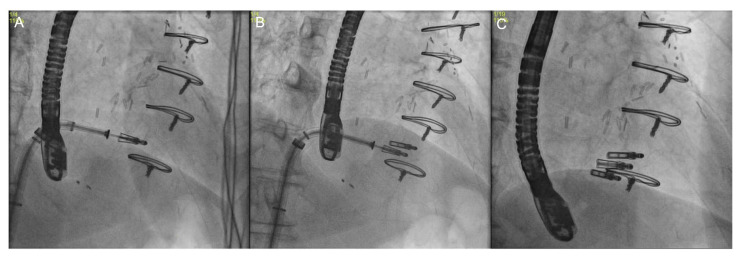
Intraprocedural fluoroscopy during transcatheter tricuspid valve edge-to-edge repair. Images show the progressive positioning of the first (**A**), second (**B**), and third (**C**) clip at the tricuspid valve level.

Fluoroscopy represents a key imaging modality for the intraprocedural guidance of transcatheter TV procedures (Figure 18), since TEE alone often provides only suboptimal TV views [62]. Despite the fact that fluoroscopy does not offer the details obtained with other imaging modalities, it is useful to provide right heart anatomical landmarks, such as the RA, RV, tricuspid annulus, interatrial septum, inferior vena cava, coronary sinus, and right coronary artery [34].

While fluoroscopy is a 2D planar imaging modality affected by parallax and requiring multiple viewing angles to obtain accurate 3D spatial data, multi-slice computed tomography (MSCT) is not affected by parallax and allows preserved spatial resolution in all imaging planes [63]. MSCT multiplanar reconstructions can therefore be used to pre-select optimal fluoroscopic viewing angles for the intraprocedural guidance of transcatheter TV interventions [43], selecting the angles that provide maximal separation among the structures of interest. The ideal projection curve (IPC) describes the TV annulus’s fluoroscopic angulations, including its en-face view and perpendicular “in-plane” views. The IPC represents the set of all fluoroscopic angles at which a given structure is displayed perpendicular to the surface. Optimal fluoroscopic angles vary on a patient-by-patient basis and their identification reduces procedure time, radiation exposure, contrast medium volume, and acute kidney injury risk, and avoids complications such as device malpositioning by facilitating coaxial device deployment [63]. Moreover, CCT data can be stored and used for intraprocedural fusion imaging with fluoroscopy [43].

Four main fluoroscopic projections are described for the right chambers. The right heart one-chamber short axis view is obtained by placing the C-arm in a left anterior oblique view or “LAO” with a variable degree of caudal angulation (average LAO 55°/CAU 15°); it shows the “enface” view of the TV seen from the RV side, mimicking the 3D echo “enface” view, and is used to navigate the catheter to the target structure; it also visualizes the trajectory of the right coronary artery and its distance from the TA, as well as the trajectory of the coronary sinus [40,62,63]. The right heart two-chambers view is obtained by placing the C-arm in a RAO projection with a variable degree of caudal angulation (average RAO 60°/CAU 50°); this view allows for the identification of the anchorage of the anterior and posterior TV leaflets to the atrioventricular junction and widens the separation between papillary muscles, avoiding subvalvular apparatus entanglement and chordae injuries with coaptation devices [40]; moreover, in this view the IVC orifice and the TV are in plane, so it is helpful to guide delivery catheters from the IVC across the TV and into the TV inflow tract. The right heart three-chambers view is obtained by orienting the C-arm in a right anterior oblique view or “RAO” with a variable degree of cranial angulation (average RAO 25°/CRA 15°); it is directed along the long axis of the RV, allows visualization of the attachments of the posterior and septal leaflets, and is used to evaluate the device’s trajectory and its co-axiality with the tricuspid annulus. Finally, the four-chambers view is obtained by orienting the C-arm in a LAO projection with marked cranial angulation (average LAO 5°/CRA 60°); it enables the view of the anterior and septal leaflet attachments and appreciates the TV annulus and atrial septum both in plane) [40,63].

### 3.4. Fusion Imaging

Interventional imagers and interventionalists usually have to mentally combine complementary data from multiple imaging modalities for intraprocedural guidance; in addition, data from different modalities are generally imaged from different perspectives and are therefore displayed in different orientations, and each modality presents different limitations [64]. Echocardiography provides a live visualization of soft tissues in 2D or 3D with anatomical and functional information and the early identification of complications, but it is subject to shadowing and blooming artifacts by the presence of catheters and devices, and it does not allow for non-cardiac anatomical landmark visualization because of the limited sector volume size. Fluoroscopy has a wider field of view and easily identifies catheters and devices, providing additional helpful information for catheter advancement and device positioning, but it is unable to visualize soft tissues, only has 2D views, and requires radiation use [64].

Fusion imaging systems combine CCT, CMR, or echocardiographic images (from TEE or ICE) with fluoroscopy, creating a multi-modalities image overlay on a single screen [31] that overcomes these limitations [17] and provides better spatial orientation, with faster and safer catheter manipulation and more accurate device orientation.

In order to create hybrid images, it is necessary to utilize compatible same-brand equipment and specialized hardware and software [64]. Static fusion imaging combines 3D datasets acquired prior to the procedure (generally from CCT, but it is possible also from CMR) with real-time intraprocedural fluoroscopy, showing anatomical structures not usually seen by fluoroscopy alone; its main limitations are misalignment with fluoroscopy due to registration errors, patient position changes and motion due to cardiorespiratory movements, and intracardiac catheter manipulation, but also changes in loading conditions between pre-procedural and intra-procedural time. Dynamic fusion imaging combines real-time acquired 3D echo and fluoroscopy data by co-registration of the echocardiography probe on the X-ray image, which provides greater accuracy since it crops soft tissue details that are not relevant for the procedure, adjusts overlay translucency to prevent fluoroscopy obscuration, and places persistent anatomic reference markers in the fluoroscopic space [31,64]. When the fluoroscopy C-arm moves, the 3D TEE image is automatically relocated based on the new X-ray projection (image-based tracking) [65]. A limitation of the current fusion system technology is the color volume overlay on the fluoroscopic monitors [31].

The benefits of fusion imaging have been shown for some structural transcatheter procedures, such as a reduced procedure time for transeptal puncture for either MitraClip or left atrial appendage closure [66], a decreased fluoroscopy time and radiation dose for atrial septum defect closure [67], and a reduced radiation dose for left atrial appendage closure [68], but there are currently no data evaluating the procedural outcomes of transcatheter tricuspid repair or replacement procedures assisted by fusion imaging, beyond individual case experience [40,69,70,71,72,73,74].

In the future, the development of more advanced fusion approaches such as a combination of CT, fluoroscopy, and echocardiography all together may further improve intraprocedural navigation.

## 4. Follow-Up of Tricuspid Transcatheter Edge-to-Edge Repair

### 4.1. Two-Dimensional and Three-Dimensional Transthoracic and Transesophageal Echocardiography

Echocardiography represents the first and key modality by which to assess the efficacy and durability of transcatheter TV repair interventions.

As T-TEER has only recently emerged as a widespread therapeutical approach, there is no standardized timing for postprocedural follow-up. However, most sites endorse outpatient evaluation at 1, 6, and 12 months after the procedure (in order to allow more initial frequent adjustments of the diuretic and HF medication regimen), followed by annual re-evaluation, in the case of procedure success [17]. The echocardiographic report should include blood pressure and drug therapy, particularly diuretic dosage, at the moment of evaluation, and the type, number, and location of the implanted leaflet device/s [17,40].

Due to the anterior position of the TV apparatus, 2D and 3D TTE echocardiography is non-inferior to TEE evaluation; usually, 2D TTE provides all the necessary information to obtain a global evaluation of the right-heart structures and follow outcomes after TV percutaneous repair. It is important to assess the grade of residual TR, to confirm the correct positioning of the device/s, and to evaluate signs of reverse remodeling of the TV apparatus (TA dimensions, leaflet tethering degree, and coaptation height and area) and of the RV, and changes in RV function [40]. The three main TTE views allowing TV visualization are the parasternal (long-axis view of the right ventricle inflow, short-axis view at the level of the aortic valve), the apical four-chamber, and the subcostal views [75].

Firstly, one should focus on the device in order to ensure appropriate leaflets insertion, arms stability, and orientation: the device must be oriented perpendicularly to the coaptation line of the selected leaflets, and this can be particularly appreciated with the 3D zoom en face view of the TV [75,76]. Doppler ultrasound imaging with both continuous waves and pulsed waves is necessary to exclude transvalvular anterograde obstruction. To minimize the respiratory flow variations, an average of five cycles is recommended, regardless of baseline cardiac rhythm [77]. In the TriValve registry, patients in the highest mean TV gradient at discharge (4.7 ± 2.0 mmHg) had similar outcomes (evaluated as a primary composite endpoint of all-cause mortality and HF hospitalizations) at 1-year follow up to patients with smaller gradients, although further investigations on higher gradients and a longer follow-up are needed [78]. In case of residual TR, it is mandatory to localize the origin of the jet and its position in respect to the device. To quantify residual TR, an integrative approach with qualitative (color flow and continuous wave Doppler signal of the TR jet) and semiquantitative (vena contracta width and hepatic vein flow pattern) methods is suggested: a large eccentric regurgitant jet reaching the posterior wall of the right atrium, with a vena contracta width > 7 mm and a holosystolic hepatic vein flow reversal, suggest a severe regurgitation. Of note, quantification with a proximal isovolumetric surface area has not been validated in this setting. Indeed, TriClip residual TR jet(s) are often sprayed in several directions [40]. Echocardiographic-assessed residual TR ≥ 3+ has been correlated with a higher risk of rehospitalization caused by HF within 1 year [36].

In case of suspicion of complications, such as device detachment, loss of leaflet insertion, leaflet tears, or active endocarditis, 2D and 3D TOE evaluation is mandatory.

Finally, post-procedural tricuspid annulus remodeling and right ventricular-pulmonary arterial (RV-PA) coupling and afterload reserve should be evaluated, as they may have a long-term prognostic relevance; a decline in the RV-PA coupling ratio (measured by dividing pulmonary artery systolic pressure by TAPSE obtained by TTE) is independently associated with a lower risk of all-cause mortality during the first year after TTVR, and is more frequent in patients with a high baseline RV-PA coupling ratio, although additional studies are needed for validation [79,80].

### 4.2. Cardiac Computed Tomography and Cardiac Magnetic Resonance

The role of cross-sectional imaging for follow-up evaluation after T-TEER still has to be defined and certainly represents a promising area for future growth [40].

Currently, cardiac CT plays a role during follow-up mainly in cases with dubious findings with the first line imaging modalities regarding tricuspid apparatus/device evaluation or the presence of potential complications [75,81]. Indeed, the presence of a tricuspid TEER device may hinder the post-procedural echocardiographic assessment of TR severity. An assessment of residual or recurrent TR by CCT is performed using the standard multi-parametric approach similar to pre-procedural evaluation.

Serial CMR assessment of the right heart chambers size, volumes, and function may also be helpful to determine responses to TV transcatheter therapy and might have a role in predicting clinical outcomes [82,83].

## 5. Future Directions: 3D Printing, Computational Models, and Artificial Intelligence

The application of new technologies to echocardiographic valvular assessment is growing and has the potential to play a pivotal role considering the fast-increasing development of these new tools, the growing volume of patient data, and the issue of clinical time restrictions.

The use of 3D printing has recently increased to reach a wide range of medical applications and, in the field of heart valve disease, it might address many of the present challenges, such as patient selection, prosthesis choice and sizing, and innovation in valve design [84]. In fact, modern technology allows for the printing of cardiac anatomy in materials that feature the characteristics of the heart structures, therefore providing better acknowledgment of the anatomy: this may not only lead to appropriate preclinical testing, but even to preoperative simulation, and therefore, the training of new structural interventionists, or postoperative retrospective procedure analyses, facilitating the learning process [84,85].

Developments in the field of artificial intelligence (AI) hold great promise too in improving the assessment and management of patients with valvular heart disease. In fact, AI may improve the acquisition and processing of echocardiographic images, as has already been demonstrated [86]. Commercial and non-commercial packages have shown good accuracy in the evaluation of valvular apparatus structures, such as leaflet segmentation, the annular perimeter, and the valve area size, for mitral [87], aortic [88], and tricuspid [89] valves. Although its routine use in clinical practice is still to be applied, and there is yet no compelling evidence on its usefulness in a TV severe regurgitation context, AI could eventually have a role in TV disease evaluation and in the identification of the most appropriate anatomy suitable for T-TEER intervention.

## Data Availability

No new data were created or analyzed in this study. Data sharing is not applicable to this article.

## Supplementary Materials

The following supporting information can be downloaded at: https://www.mdpi.com/article/10.3390/jcm12103384/s1, Video S1: 2D Transesophageal echocardiogram transgastric view short axis 30° documenting all three tricuspid valve leaflets.
